# 4,4’-trimethylenedipiperidine as a nitrogen heterocycle solvent and/or catalyst: Liquid phase tandem Knoevenagel–Michael condensation

**DOI:** 10.3906/kim-2010-41

**Published:** 2021-02-17

**Authors:** Lia ZAHARANI, Nader GHAFFARI KHALIGH, Hayede GORJIAN, Mohd RAFIE JOHAN

**Affiliations:** 1 Nanotechnology and Catalysis Research Centre, Institute for Advanced Studies, University of Malaya, Kuala Lumpur Malaysia; 2 Department of Food Science and Technology, Sari Agricultural Sciences and Natural Resources University, Sari Iran

**Keywords:** Homogeneous catalysis, heterocycles, multicomponent reactions, waste prevention

## Abstract

Liquid phase tandem Knoevenagel–Michael condensation of various aromatic and heteroaromatic aldehydes with barbituric acid or 2-thiobarbituric acid and malononitrile was studied in a one-pot three-component reaction. For the first time, TMDP was employed as a safe and efficient solvent and/or catalyst in the liquid and aqueous ethanol medium, respectively, for the practical and eco-friendly Knoevenagel–Michael condensation. The reactions were carried out by using greener procedures, including a) the use of TMDP as an N-heterocycle organocatalyst in a green medium including water and ethanol (1:1 v/v) at reflux temperature, and b) the use of TMDP as a dual solvent-catalyst at 65 °C in the absence of any solvent. High to excellent yields of the desired pyrano[2,3- *d* ]pyrimidinones were obtained under the two earlier mentioned conditions. The current methodologies have advantages, including (a) avoiding hazardous, toxic, volatile, and flammable materials and solvents, (b) avoiding tedious processes, harsh conditions, and multiple steps for the preparation of catalysts, (c) using a less toxic and noncorrosive catalyst, (d) minimizing hazardous waste generation and simple workup process, and (e) high recyclability of TMDP. Another important result of this work is that the TMDP can be a promising alternative for toxic, volatile, and flammable base reagents such as piperidine and triethylamine in liquid phase organic syntheses owing to its unique properties such as being less toxic, nonflammable, and nonvolatile, and having a low melting point, broad liquid range temperature, high thermal stability, and safe handling and storage.

## 1. Introduction

Catalysts play a vital role in both academic and industrial processes. They are widely employed in organic chemistry. Solvents can also serve one or more functions in the chemical procedures. The solvent type and polarity can affect the selectivity, reactivity, rate, and yield of reactions. The current trend in organic reactions is to develop greener and eco-friendly methods, as well as to direct the activities towards sustainability and investigate the catalytic efficiency under realistic conditions regarding temperature and pressure. Thus, it is highly demanding to carry out a multicomponent reaction (MCR) in a green solvent using an efficient catalyst without particular facilities and precautions. Organocatalyts have been employed in MCRs due to passive interactions like hydrophobic, Van der Waals, and electrostatic interactions along with dynamic interactions viz. hydrogen bonding of the substrates with the active sites of organocatalyst [1]. An ideal catalyst should be efficient, low-loading, inexpensive, simply separable, less or nontoxic, and highly recyclable and should offer easy handling, safe storage, etc. The commercially available catalysts can save time, energy, and cost in most circumstances. 

Different classes of nitrogen heterocycles have been prepared through MCRs using green, unconventional, and selective conditions [2–5]. The pyrimidine moiety is one of the abundant heterocycles in medicine and drug researches [6]. Pyranopyrimidinones have attracted much attention owing to their broad range of pharmaceutical and therapeutic properties. The promising biological activities of these nitrogen heterocycles have also been reported in the literature [7–16]. Piperidine has been widely used as an N-heterocycle organocatalyst for the synthesis of heterocycle compounds through a tandem Knoevenagel-–Michael condensation reaction [17–21]. In addition, the synthesis of pyrano[2,3‐ *d* ]pyrimidine‐2,4‐dione derivatives using trimethylamine in ethanol under reflux conditions has been reported [22]. Due to the broad range of biological activities of pyrano[2,3- *d* ]pyrimidinones, different methods and various types of catalysts have been reported for their synthesis, as indicated by the number of publications [23–30]. Some protocols have certain drawbacks, along with their advantages. Very often, there are two or more preparative steps for the fabrication of the catalysts, which can raise the cost and may involve the use of toxic and volatile solvents. Some other methods require a tedious work-up along with the several times washing and rinsing the products or catalysts, which leads to generating toxic and hazardous wastes. Hence, there is a high demand to develop greener protocols, which employ nontoxic, nonflammable, and nonvolatile solvents along with inexpensive, easily separated, and recyclable catalysts. 

4,4’-Trimethylenedipiperidine (TMDP) is equivalent to two piperidines linked by a three-carbon spacer which is able to act as an acceptor-donner hydrogen bond. It is commercially available, less toxic, easy-to-use, as well as stable up to high temperatures at a nitrogen atmosphere without decomposition, and shows good solubility in water. TMDP has a low melting point (52.3 °C) and a broad liquid range temperature (~280 °C). Based on the unique properties of TMDP and its successful catalytic applications in some organic syntheses [31–33], we decided to investigate the potential of TMDP as an *N* -heterocycle catalyst in aqueous ethanol and as a dual solvent-catalyst in its liquid state at 65 °C, for the preparation of pyrano[2,3- *d* ]pyrimidinones via a one-pot three-component reaction. 

## 2. Materials and methods

### 2.1. General

The reagents, solvents, and chemical compounds were of analytical grade and provided from Merck, Sigma Aldrich, Alfa Aesar Chemical Companies, and used without further purification. The ^1^H NMR spectra were recorded with a Bruker Avance 400 MHz instrument. All chemical shifts are quoted in parts per million (ppm) relative to TMS using DMSO- *d*_*6*_ as a deuterated solvent. Melting points were recorded on a Büchi B-545 apparatus in open capillary tubes. Microanalyses were performed on a Perkin-Elmer 240-B microanalyzer.

### 2.2. The typical procedure for the preparation of pyrano[2,3-d]pyrimidinone using TMDP as a catalyst in a mixed solvent containing water and ethanol (1:1 v/v)

A variety of aldehydes (0.5 mmol) were mixed with barbituric acid (65 mg, ~0.5 mmol) or 2-thiobarbituric acid (73 mg, ~0.5 mmol), malononitrile (33.5 mg, ~0.5 mmol), and TMDP (20 mg, 0.1 mmol) in a mixture solvent of water/ethanol [1:1 v/v] (1.0 mL). The mixture was heated and stirred at 85 °C for the appropriate time. After the consumption of the reactants (monitored by TLC), the deionized water (2.0 mL) was poured into the flask and the reaction mixture was stirred for 5 min. The precipitated product was separated by simple filtration and rinsed with cold deionized water (3 × 2 mL). The pure product was isolated after drying with no requirement for column chromatography or recrystallization (monitored by ^1^H NMR). After that, the filtrated solution was concentrated under a partial vacuum by a rotary evaporator. After adding an appropriate amount of ethanol to the aqueous solution of TMDP, it was reused in the next run without any washing, drying, or purification. 

### 2.3. The typical procedure for the preparation of pyrano[2,3-d]pyrimidinones using TMDP as dual solvent-catalyst in the liquid state

The mixture of aldehydes (0.5 mmol), barbituric acid (65 mg, ~0.5 mmol), or 2-thiobarbituric acid (73 mg, ~0.5 mmol), and malononitrile (33.5 mg, ~0.5 mmol) were stirred in TMDP (125 mg) at 65 °C. The reaction mixture was diluted by deionized water (0.5 mL) after completion of the reaction (monitored by TLC). The crude product and catalyst were separated with simple filtration. The crude products were obtained using the procedure mentioned earlier. The solvent was removed from the aqueous solution of TMDP with a rotary evaporator under vacuum, and the recovered TMDP was reused in the next runs with no more rinse, drying, or purification. The purity of products was approved by comparing the melting point, ^1^H NMR, and elemental analysis with those of the known compounds reported in the literature (see Supplementary Information) [28–30]. 

## 3. Results and discussion

### 3.1. The Synthesis of pyrano[2,3-d]pyrimidinones derivatives in the presence of TMDP as a catalyst or dual solvent-catalyst

Initially, the condensation of three model reactants viz. 4-chlorobenzaldehyde (1a), barbituric acid, and malononitrile was investigated in different conditions to find the optimal conditions (Table 1). The equimolar model reactants were mixed and stirred at room or reflux temperature in water as green solvent for 2 h under catalyst-free conditions (Table 1, entries 1 and 2). The unreacted 4-chlorobenzaldehyde was observed on TLC at room and reflux temperatures and the reaction was not completed after 2 h. The addition of a catalytic amount of TMDP caused a remarkable rise in the yield of 7-amino-6-cyano-5-(4-chlorophenyl)-4-oxo-5 *H* -pyrano[2,3- *d* ]pyrimidinone (2a) at room and reflux temperatures (Table 1, entries 3-5). Regarding the limited solubility of substrates in water, the next experiments were conducted in a volume ratio of 1:1 of water and ethanol, which led to an improvement in the yield of 2a (Table 1, entry 6). The model reaction produced the same yield when the reaction time was shortened to 60 min (Table 1, entry 7). Then, the amount of TMDP was increased to 100 mg, which caused a negligible rise in the yield of 2a (Table 1, entry 8). The above results exhibited that this three-component reaction required a catalyst and higher temperature due to its high activation energy. 

**Table 1:** Influence the different parameters on the yield of the model reaction product.a

Entry	Amount ofTMDP (mg)	Solvent	Temp. (°C)	Time (min)	Yield (%)b
1	-	H2O	r.t.	120	11
2	-	H2O	Reflux (98 °C)	120	32
3	40	H2O	r.t.	120	41
4	40	H2O	Reflux (98 °C)	120	59
5	80	H2O	Reflux (98 °C)	120	70
6	80	H2O/EtOH (1:1 v/v)	Reflux (85 °C)	120	88
7	80	H2O/EtOH (1:1 v/v)	Reflux (85 °C)	60	88
8	160	H2O/EtOH (1:1 v/v)	Reflux (85 °C)	60	92
9	500	Liquefied TMDP	65	120	92
10	500	Liquefied TMDP	65	60	92
11	500	Liquefied TMDP	65	40	77

a Reaction conditions: 4-chlorobenzaldehyde (285.0 mg, ~2.0 mmol), barbituric acid (258.8 mg, ~2.0 mmol), malononitrile (133.5 mg, ~2.0 mmol), solvent (2 mL).b The reaction mixture was triturated in water and the product was purified by crystallization from ethanol.

In our previous work, it was indicated that TMDP has a low melting point (52.3 °C) and a high boiling point (332.5 °C) [30]. Hence, TMDP has a wider liquid range (~280 °C) than water and ethanol. Furthermore, the TMDP has two Lewis base sites, and it can simultaneously act as both hydrogen bond acceptor and donor. In the next experiments, the model reaction was conducted at 65 °C, where TMDP changed into its liquid state, and the mixture of reactants was easily stirred in the liquefied TMDP (Table 1, entries 7–9). The highest yield was observed for the length of time 60 min (Table 1, entry 10).

**Scheme 1:**
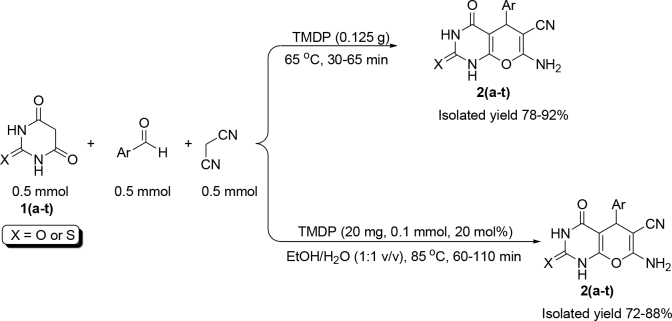
Two new protocols for the synthesis of pyrano[2,3-d]pyrimidinones using TMDP.

Entries 7 and 10 (shown in bold) in Table 1 were selected as the optimized reaction conditions. Then, the substrate scope of the current protocols was investigated to condense various aromatic and hetero-aromatic aldehydes with barbituric acid and malononitrile under optimized reaction conditions (Scheme 1).

The results showed that the corresponding pyrano[2,3- *d* ]pyrimidinones could be isolated in good to high yield in both optimized conditions (Table 2). The desired product was obtained in high yield with the current protocol when 2-furaldehyde, a sensitive hetero-aryl aldehyde to acid medium, was employed under optimal conditions. The nature and position of substituents exhibited no significant effect in the reaction times and yields within the experimental error. As one can see in Table 2, the condensation of thiobarbituric acid with aldehydes and malononitrile required longer reaction times. 

**Table 2:** The preparation of pyrano[2,3-d]pyrimidinone derivatives using TMDP as organocatalyst under optimal conditions.a

Entry	1(a-j)	X	2(a-j)	Method Ab		Method Bc		Melting point (°C)	
				Time/min	Yield/%d	Time/min	Yield/%d	Found	Reported [Ref.]
1	4-Cl-C6H4-	O	2a	30	92	60	88	240-241	242-243 [29]
2	4-Cl-C6H4-	S	2b	55	90	85	85	>300	>300 [29]
3	C6H5-	O	2c	30	82	60	80	225-226	222-224 [29]
4	C6H5-	S	2d	40	85	75	82	224-225	223-224 [29]
5	3-Cl-C6H4-	O	2e	30	78	60	75	238-239	240-241 [30]
6	3-Cl-C6H4-	S	2f	35	80	80	78	234-235	237-238 [30]
7	2,3-Cl2-C6H3-	O	2g	30	82	60	78	238-239	240-242 [30]
8	2,3-Cl2-C6H3-	S	2h	55	86	90	81	254-256	257-258 [30]
9	2,4-Cl2-C6H3-	O	2i	30	85	60	83	239-240	241-242 [30]
10	2,4-Cl2-C6H3-	S	2j	65	82	110	80	237-238	238.5-239.5 [30]
11	4-Br-C6H4-	O	2k	30	85	60	85	227-228	230-231 [30]
12	4-Br-C6H4-	S	2l	50	87	85	87	239 (dec.)	236 (dec.) [30]
13	4-NO2-C6H4-	O	2m	30	83	60	83	240-241	239-240 [30]
14	4-NO2-C6H4-	S	2n	55	85	100	85	236-237	235-236 [30]
15	3-NO2-C6H4-	O	2o	30	82	60	82	271-272	268-270 [30]
16	3-NO2-C6H4-	S	2p	40	81	85	81	230-231	233-234 [30]
17	4-CF3-C6H4-	O	2q	30	78	60	72	248-249	250-251 [30]
18	4-CF3-C6H4-	S	2r	60	82	95	79	238-239	239-240 [30]
19	2-Furfuryl	O	2s	30	83	60	82	278-279	280-282 [29]
20	2-Furfuryl	S	2t	45	84	80	83	282-283	281-282 [29]

a Reaction conditions: various aldehydes 1(a-t) (0.5 mmol), barbituric acid (65 mg, ~0.5 mmol) or 2-thiobarbituric acid (73 mg, ~0.5 mmol), malononitrile (33.5 mg, ~0.5 mmol) b The liquid state of TMDP (125 mg), reaction temperature (65 °C) c 20 mol% of TMDP (20 mg, 0.1 mmol), two-mixed solvent (ethanol and water, 1:1 v/v) (0.5 mL), reaction temperature (85 °C) d Isolated yield.

 To study the superiority of TMDP in comparison to piperidine, the model reaction was conducted by using 40 µL (~20 mol%) and 80 µL (~40 mol%) of piperidine in 2.0 mL of an equal volume of the two-mixed solvent of ethanol and water under reflux conditions. The corresponding product was afforded in 41% and 76% yields, respectively, after 60 min. Moreover, the model reaction was performed into 0.5 g of piperidine at 65 °C for 1 h, and 2a was obtained in 86% yield. The above results demonstrated that TMDP was superior to two equivalents of the piperidine ring. As previously reported, the TMDP is preferred in comparison to piperidine because piperidine is a volatile, flammable, highly toxic, and unsafe-to-handle liquid. Furthermore, piperidine is used in the preparation of illegal psychotropic drugs, and this fact causes a limited availability of piperidine in some countries [34].

**Scheme 2:**
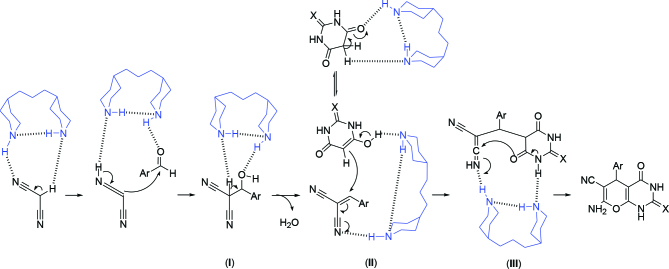
A possible mechanism for the synthesis of pyrano[2,3-d]pyrimidines.

Although our group is investigating the detailed mechanism of the model reaction in the liquid phase of TMDP, a proposed route is illustrated in Scheme 2. Initially, the malononitrile and aryl aldehyde can be activated via H-bond formation and hydrogen transformation with TMDP, which facilitates the Knoevenagel condensation. The dehydration of intermediate **I** gives olefin **II** . TMDP also activates barbituric acid or thiobarbituric acid which attacks olefin **II** to give the final product, after hydrogen transfer, tautomerization, and hydrolysis of intermediate **III** .

Finally, the condensation of model reactants was carried out on a large scale as an industrial application. A 100-mL bottom round flask was charged with 12.50 g of TMDP and heated up to 65 °C. Then, 7.1 g of 4-chlorobenzaldehyde, 6.5 g of barbituric acid, and 3.4 g of malononitrile were slowly added to the liquefied TMDP, with continuous stirring by a mechanical stirrer. After 1 h, 15 mL of deionized water was poured into the reaction mixture, and the product was filtered and rinsed several times with deionized water (3 × 5 mL). The crude product was purified through crystallization from the hot ethanol (10 mL). The pure product was isolated at 12.8 g (yield ~81%). Water was removed from the filtrate by a rotary evaporator, and the obtained TMDP was utilized for the small-scale reactions.

The reusing results exhibited that the TMDP could be reused for at least five successive runs, and the isolated yields of the corresponding product were in the range of 88-–81%. An average of 3.8 wt. % TMDP loss was observed during subsequent runs. The ^1^H NMR analysis of fresh and recycled TMDP (5th run) showed no significant change in the chemical structure. The studies showed negligible leaching of TMDP into the product of 2a (LC-MS analysis).

Table 3 shows that our results are compared with some published methods in the literature [18–26]. Due to drawbacks and limitations, many of them could not be applied in industrial or academic procedures. Some disadvantages can be cited as the fabrication of catalysts using hazardous reactants and toxic, flammable, and volatile solvents and reagents, which requires a long time and tedious and multiple steps. Each step requires several rinses and washing, generating hazardous waste, and some catalysts require an activation step before their application (Table 3, entries 1 and 8). Separation of nanocatalysts can often be conducted via centrifuge, and their leakage can cause environmental issues (Table 3, entry 8). Furthermore, some catalysts cannot practically be recycled (Table 3, entries 3– 7). Some methods have a limited substrate scope (Table 3, entries 3–7) or use a fatal, volatile, and flammable liquid as a promoter (Table 3, entry 2). 

**Table 3:** A comparative table for the synthesis of pyrano[2,3-d]pyrimidines.

Entry	Catalyst	Loading (mol %)	Solvent	Conditions	Reactiontime (min)	Yield(%)	Ref.
1	Formamidine sulfonic acid (FSA) stabilized on silica-coated Fe3O4magnetic nanoparticles	20 mg	H2O	50 °C	6 h	73–91	23
2	Dibutylamine (DBA)	20	EtOH/H2O(1:1 v/v)	Reflux temp.	43–129	83–94	24
3	1,4-diazabicyclo[2.2.2]octane(DABCO)	10	EtOH/H2O(1:1 v/v)	Room temp.	120	83–96	25
4	KAl(SO4)2.12H2O (alum)	10	H2O	80 °C	30–45	80–90	26
5	-	-	-	Ball-milling(20–25 Hz), 96 °C	30–90	94–99	27
6	L-Proline	5	EtOH/H2O(1:1 v/v)	Room temp.	30–90	68–86	35
7	(NH4)2HPO4	10	EtOH/H2O(1:1 v/v)	Room temp.	120	70–90	29
8	sulfonic acid nanoporous silica(SBA-Pr-SO3H)	10 mg	-	100 °C for activation, 140 °C for reaction	5–45	30–90	36
9	TMDP	20	EtOH/H2O(1:1 v/v)	85 °C	60–110	72–88	This work
	TMDP	0.125 g	-	65 °C	30–65	78–92	

In conclusion, a new application of TMDP was demonstrated to promote a one-pot three-component reaction as a) a dual solvent-catalyst in its liquid state and b) a catalyst in a mixture of green solvents. The pyrano[2,3- *d* ]pyrimidinones were isolated in high to excellent yields. The advantages of the current methodologies are (a) safe and greener conditions, (b) simple separation of catalyst or solvent/catalyst and desired products, (c) minimized hazardous waste generation, and (d) high recyclability of organocatalyst. The unique features of TMDP, such as being commercially available, having wide liquid range, bearing Lewis base sites and hydrogen bond acceptor-donner groups, safe handling and storage, make it a promising organocatalyst for organic synthesis. Furthermore, the TMDP can be a safe alternative for toxic, flammable, and volatile organic base catalysts.
